# IT’S ACTUALLY WORSE THAN IT LOOKS: IDENTIFYING ABUSE-RELATED INJURIES AMONG APS CLIENTS

**DOI:** 10.1093/geroni/igz038.2131

**Published:** 2019-11-08

**Authors:** Jeanine Yonashiro-Cho, Zach Gassoumis, Kathleen Wilber, Diana Homeier

**Affiliations:** 1 Keck School of Medicine of USC, Alhambra, California, United States; 2 Leonard Davis School of Gerontology, University of Southern California, Los Angeles, California, United States; 3 Keck School of Medicine of USC, University of Southern California, Los Angeles, California, United States

## Abstract

While recent work has described elder abuse injuries seen in medical contexts, most abuse determinations are made by community-based health and social services practitioners in the field. Little is known about the types of injuries present among victims who do not seek medical care. The purpose of this study was to identify and describe injuries more likely to occur through abuse, rather than accidental injury, among older adults seen in non-medical settings. An observational, matched-comparison group design was used to compare findings among physically abused APS clients (n=61) with those from non-abused older adults (n=104) seeking usual-care in a Geriatrics clinic. Forensic nurse examiners conducted full-body examinations of subjects and collected data on injury diagnoses, locations, and characteristics. Descriptive statistics and bivariate tests of association were used to analyze differences in injury presentation between groups. Though 21.8% of APS clients had no observable injuries upon examination, as a group, they were more likely than non-abused elders to be injured (p<0.05) and had more injuries present (p<0.01). Abuse victims were also more likely to have at least one upper extremity abrasion (p<0.05) or a diagnosis of ecchymosis (p<0.01), swelling (p<0.05) or tenderness (p<0.05) in the head, neck, or maxillofacial region. Because physical abuse may not result in injury to victims, screening protocols are needed to improve abuse detection. The presence of injuries among older adults at-risk for abuse warrant further evaluation or queries from medical and social service providers, regardless of injury severity.

